# Triazole‐bearing oligo(ethylene glycol)‐strapped zinc porphyrins as dual mode ion‐binding receptors

**DOI:** 10.1002/smo.20220004

**Published:** 2023-03-20

**Authors:** Jeong Heon Lee, Kyeong‐Im Hong, Woo Hyeok Choi, Younghun Kim, Woo‐Dong Jang

**Affiliations:** ^1^ Department of Chemistry Yonsei University Seodaemun‐Gu Seoul Korea

**Keywords:** crown ether, ion receptor, macrocycle, porphyrin, triazole

## Abstract

A series of triazole‐bearing oligo(ethylene glycol)‐strapped zinc porphyrins (**P**_**Zn**_ **4EG** and **P**_**Zn**_ **5EG**) have been prepared as artificial ion‐binding receptors. Both **P**_**Zn**_ **4EG** and **P**_**Zn**_ **5EG** exhibit UV/Vis absorption changes in a solvent‐dependent manner upon titration with halides and alkali metal ions. In tetrahydrofuran, both **P**_**Zn**_ **4EG** and **P**_**Zn**_ **5EG** exhibit spectral shifts upon the addition of halide ions through axial coordination, whereas the absorption does not change upon the addition of alkali metal ions. On the other hand, in 10% MeCN/CHCl_3_, the UV/Vis absorption spectra of both **P**_**Zn**_ **4EG** and **P**_**Zn**_ **5EG** do not change upon the addition of an excess amount of halide ions; however, **P**_**Zn**_ **4EG** and **P**_**Zn**_ **5EG** show selective alkali metal ion‐binding properties in this solvent. **P**_**Zn**_ **4EG** and **P**_**Zn**_ **5EG** display dramatic color changes and spectral shifts upon the addition of Li^+^ and K^+^, respectively. Because of the dramatic color changes that occurred upon binding, the determination of alkali metal ions would be possible.

## Graphical abstract

Triazole‐bearing oligo(ethylene glycol)‐strapped zinc porphyrins show dual mode ion‐binding behavior with solvent‐dependent selectivity between halides and alkali metal ions.

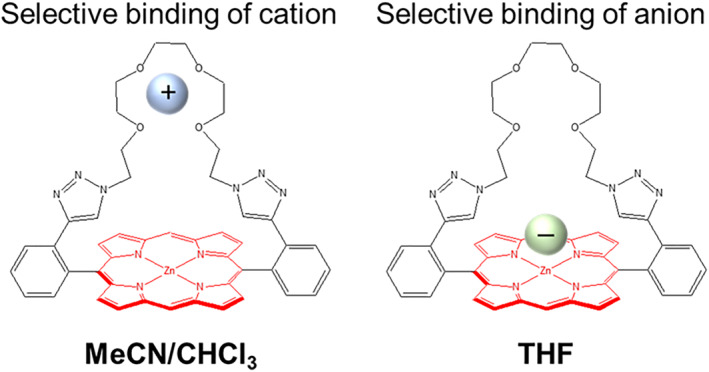

## 1 INTRODUCTION

**SCHEME 1:**
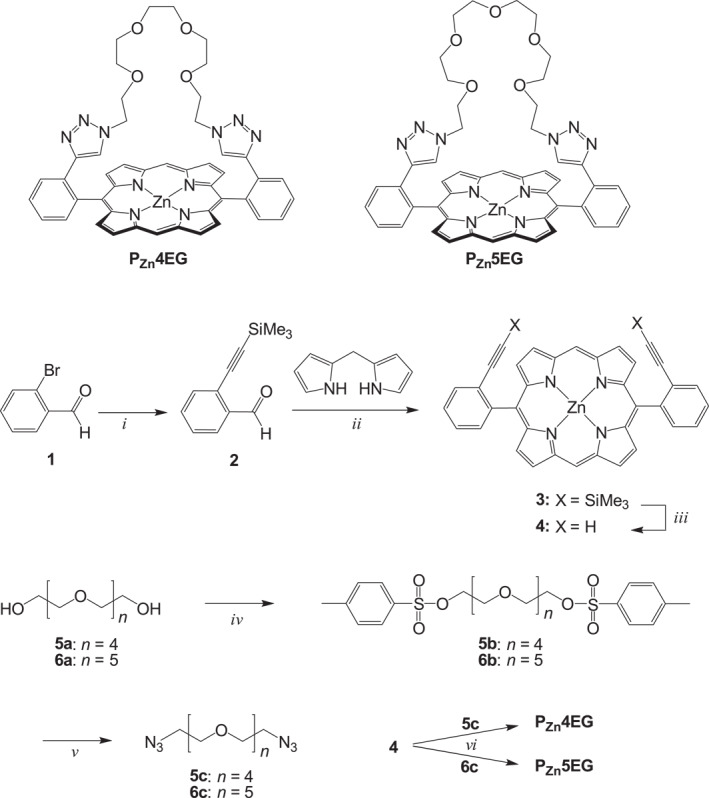
Structures of **P**_**Zn**_ **4EG** and **P**_**Zn**_ **5EG** and corresponding synthetic procedures. Reactants and conditions: (*i*) TMS‐acetylene, Pd(PPh_3_)_2_Cl_2_, CuI, N_2_, triethylamine, reflux, 12 h; (*ii*) CF_3_CO_2_H, triethylamine, *p*‐chloranil, Zn(OAc)_2_, CH_2_Cl_2_, 5 h; (*iii*) *n*‐Bu_4_NF, THF; (*iv*) *p*‐TsCl, KOH, CH_2_Cl_2_, 0°C, 12 h; (*v*) NaN_3_, EtOH, 48 h; and *vi*) [Cu(CH_3_CN)_4_]PF_6_, N_2_, DMF, 70°C, 72 h.

Selective ion‐binding receptors are of great interest in biological and industrial applications.^[1]^ Macrocyclic compounds are useful motifs for the design of ion‐binding artificial receptors.^[2]^ For example, calix[4]pyrrole derivatives are well‐known artificial anion‐binding receptors that can accommodate various anionic species as guest compounds through multiple hydrogen bonding interactions.[^1^, ^3^] As another example, Flood et al. developed a series of triazolophane macrocycles, which can accommodate halide ions via multiple C–H∙∙∙X^−^ hydrogen bonds.^[4]^ Crown ethers are representative examples of macrocyclic hosts used for the binding of alkali metal ions.^[5]^ Depending on the size of the macrocycle, crown ethers exhibit high selectivity toward metal ions.^[6]^ We recently reported a calix[4]pyrrole bearing triazole groups that acted as a strong anion binding receptor, showing strong binding affinity toward halide anions through the cooperative effect of pyrrolic N–H∙∙∙X‐ and triazole C–H∙∙∙X^−^ hydrogen bonds. Moreover, lithium salts have been successfully extracted from aqueous media upon the introduction of an oligo(ethylene glycol) strap.^[7]^ In addition, picket fence‐type triazole‐bearing zinc porphyrins exhibit strong binding affinities toward various anionic species through the cooperative effect of axial coordination and C–H∙∙∙X^−^ hydrogen bonding interactions.^[8]^ In this study, we designed a series of triazole‐bearing porphyrins bearing an oligo(ethylene glycol) strap (**P**_**Zn**_ **4EG** and **P**_**Zn**_ **5EG**; Scheme 1) as artificial ion binding receptors, which exhibit colorimetric changes upon the formation of host–guest complexes with anionic and cationic species.

## 2 RESULTS AND DISCUSSION

### 2.1 Synthesis of triazole‐bearing oligo(ethylene glycol)‐strapped zinc porphyrins

The syntheses of **P**_**Zn**_ **4EG** and **P**_**Zn**_ **5EG** are illustrated in Scheme 1. Trimethylsilyl (TMS)‐acetylene was introduced to 2‐bromobenzaldehyde (**1**) via a Sonogashira coupling reaction to give **2**. The porphyrin precursor was synthesized by an acid‐catalyzed condensation reaction between 2 and dipyrrolomethane and then successively oxidized using *p*‐chloranil as an oxidant. Zinc ions were introduced into the porphyrin core using zinc acetate to obtain **3**. The TMS groups in **3** were removed upon treatment with tetrabutylammonium fluoride (*n*‐Bu_4_NF) to produce **4**. The terminal hydroxy groups in the oligo(ethylene glycol) compounds (**5a** and **6a**) were converted into p‐toluene sulfonyl groups, which were further converted into azide groups to obtain azide–functionalized oligo(ethylene glycol)s **5c** and **6c**, respectively. Finally, a Cu(I)‐catalyzed cycloaddition reaction between **4** and azide‐functionalized oligo(ethylene glycol)s (**5c** and **6c**) was conducted to obtain triazole‐bearing oligo(ethylene glycol)‐strapped zinc porphyrins **P**_**Zn**_ **4EG** and **P**_**Zn**_ **5EG**, respectively. The as‐synthesized porphyrin derivatives were characterized using ^1^H and ^13^C NMR spectroscopy, MALDI‐TOF‐MS, and single X‐ray crystallography.

### 2.2 Binding of halides

**FIGURE 1:**
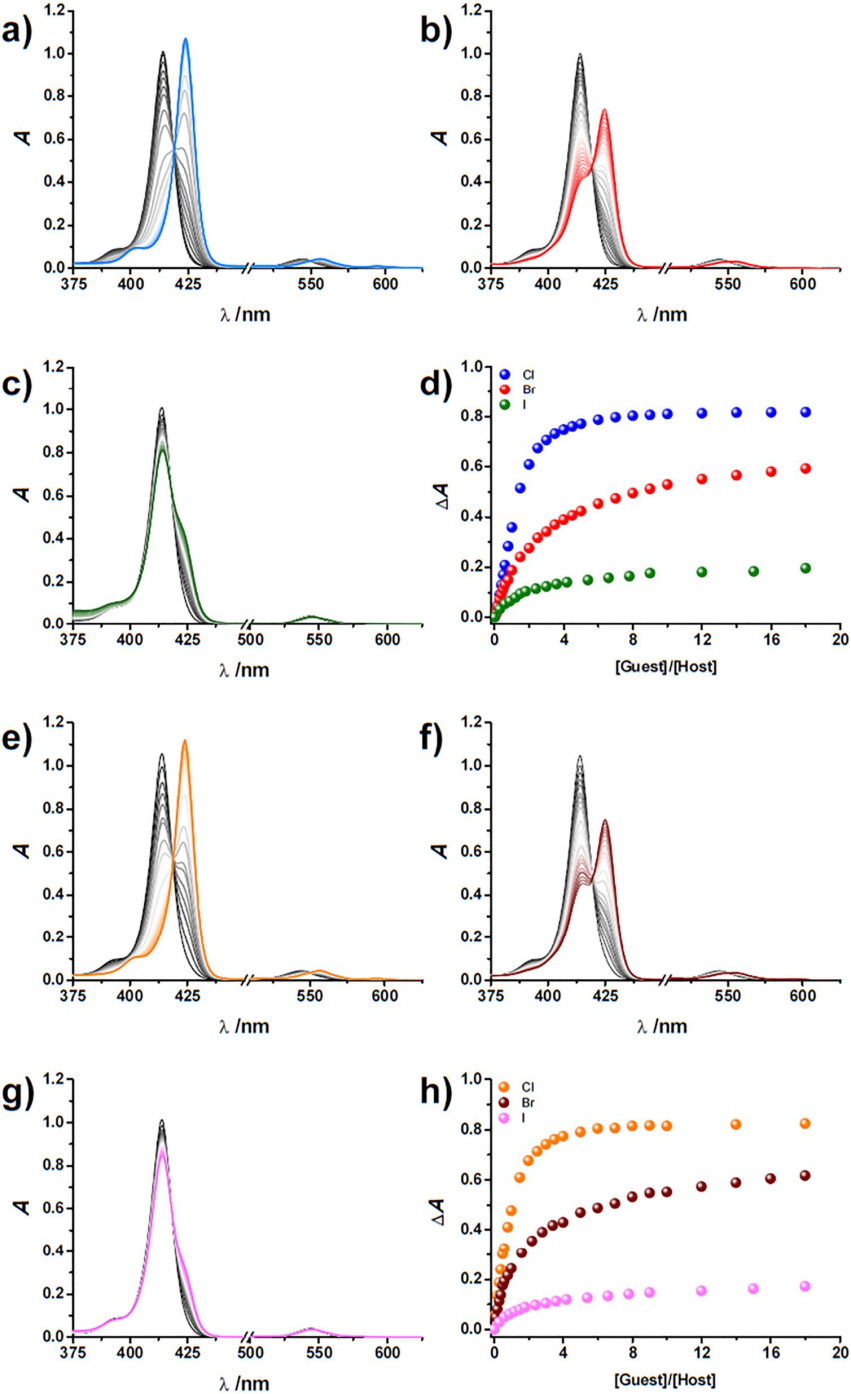
Spectral changes of **P**_**Zn**_ **4EG** (a)–(c) and **P**_**Zn**_ **5EG** (e)–(g) upon the addition of halides. (a), (e) Cl^−^, (b), (f) Br^−^, and (c), (g) I^−^. Binding isotherms monitored at the absorption maxima observed for (d) **P**_**Zn**_ **4EG** and (h) **P**_**Zn**_ **5EG** upon the addition of halides ([Host] = 2.5 µM, [Guest] = 0–50 µM).

As previously reported, triazole‐bearing picket fence‐type porphyrins exhibit strong binding affinities toward halides through the cooperative effect of C–H∙∙∙X^−^ hydrogen bonding and axial coordination interactions.^[8]^ The UV/Vis absorption spectral change of **P**_**Zn**_ **4EG** and **P**_**Zn**_ **5EG** in THF and CHCl_3_ including 10% MeCN were investigated upon the addition of halides to obtain information on the mechanism of host–guest complex formation. In THF, **P**_**Zn**_ **4EG**, and **P**_**Zn**_ **5EG** show strong Soret and Q band absorptions at 414 and 540 nm, respectively. Upon the addition of halide ions, as the form of their tetrabutylammonium (TBA) salts, the **P**_**Zn**_ **4EG** and **P**_**Zn**_ **5EG** exhibit a spectral shift with clear isosbestic points, indicating the formation of host–guest complexes via axial ligation. When Cl^−^ and Br^−^ were added to **P**_**Zn**_ **4EG** and **P**_**Zn**_ **5EG**, the wavelengths of the absorption maxima (*λ*_max_) were shifted to 424 and 425 nm, respectively. On the other hand, the addition of I^−^ to **P**_**Zn**_ **4EG** and **P**_**Zn**_ **5EG** caused the generation of a shoulder peak in the red‐shifted region along with a decrease in the intensity of the *λ*_max_ peaks (Figure 1). A continuous variation method (Job's plot) indicated the formation of 1:1 host–guest complexes between both **P**_**Zn**_ **4EG** and **P**_**Zn**_ **5EG** and all three halides (Figure S1). The binding isotherm monitored at *λ*_max_ indicates that the binding affinity toward halides observed for **P**_**Zn**_ **4EG** and **P**_**Zn**_ **5EG** was in the order of I^−^ < Br^−^ < Cl^−^. The association constants for **P**_**Zn**_ **4EG** and **P**_**Zn**_ **5EG** toward halides were estimated by nonlinear curve fitting analysis of the spectral changes utilizing HypSpec software (Table 1). The binding of halides to **P**_**Zn**_ **4EG** and **P**_**Zn**_ **5EG** was also investigated in 10% MeCN/CHCl_3_, in which Soret and Q band absorptions of both **P**_**Zn**_ **4EG** and **P**_**Zn**_ **5EG** appeared at 415.5 and 550 nm, respectively. Although each halide was added to a large excess of **P**_**Zn**_ **4EG** and **P**_**Zn**_ **5EG** in 10% MeCN/CHCl_3_, spectral changes were not observed for both **P**_**Zn**_ **4EG** and **P**_**Zn**_ **5EG**, indicating that the halide ions do not bind to **P**_**Zn**_ **4EG** and **P**_**Zn**_ **5EG** under these solvent conditions (Figure S2).

**TABLE 1:** Association constants obtained for **P**_**Zn**_ **4EG** and **P**_**Zn**_ **5EG** toward halides.

Host	Guest	*K* (M^−1^)
**P**_Zn_ **4EG**	Cl^−^	8.3 × 10^5^
	Br^−^	2.0 × 10^5^
	I^−^	1.2 × 10^5^
**P**_Zn_ **5EG**	Cl^−^	1.1 × 10^6^
	Br^−^	2.6 × 10^5^
	I^−^	1.3 × 10^5^

### 2.3 Alkali metal ion bindings

**FIGURE 2:**
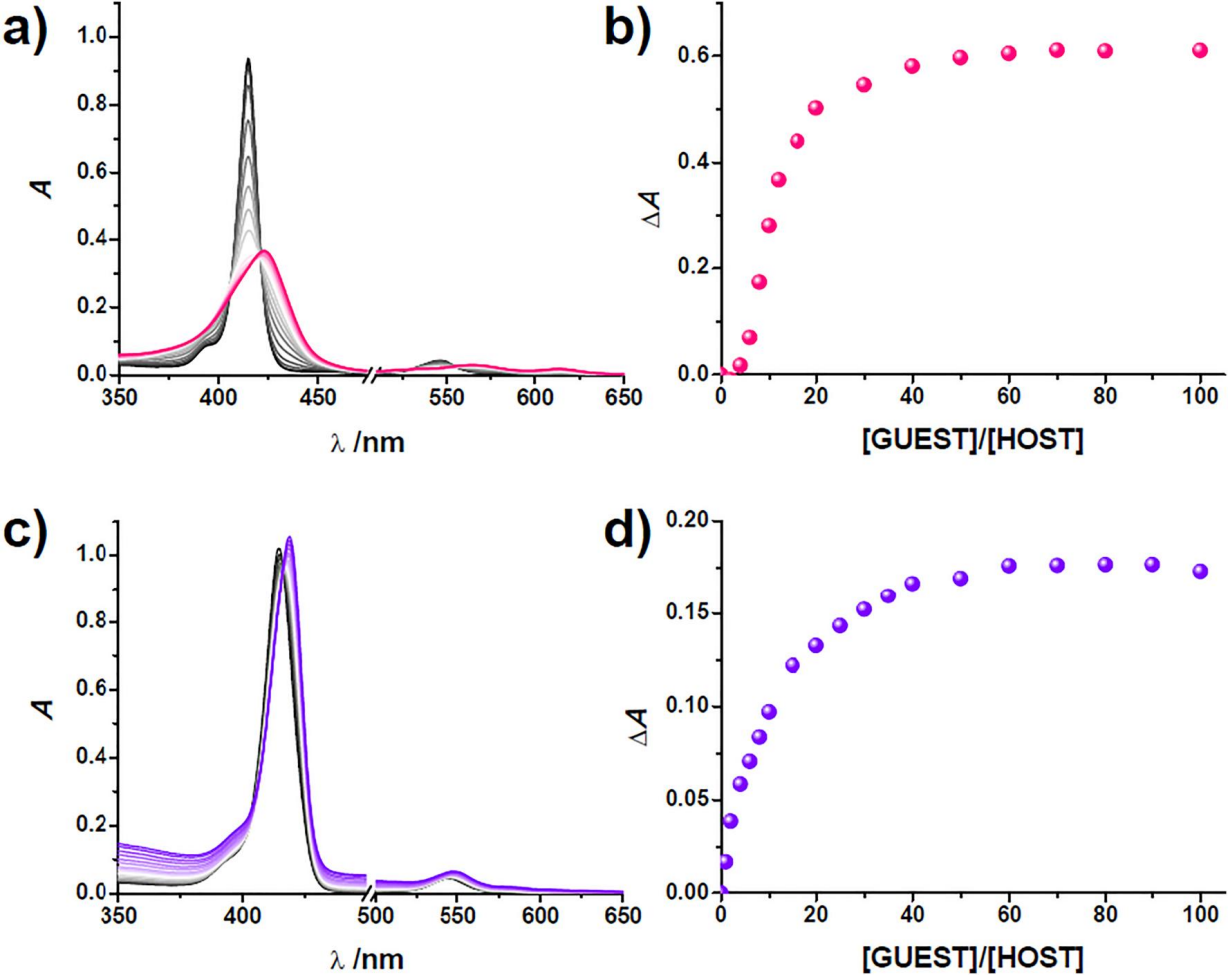
Spectroscopic titration of **P**_**Zn**_ **4EG** and **P**_**Zn**_ **5EG** with Li^+^ and K^+^, respectively. The spectral changes observed for (a) **P**_**Zn**_ **4EG** and (c) **P**_**Zn**_ **5EG**. The binding isotherms obtained for (b) **P**_**Zn**_ **4EG** and (d) **P**_**Zn**_ **5EG** ([Host] = 2.5 µM, [Guest] = 0–250 µM).

It is well known that crown ethers are excellent host compounds for binding alkali metal ions in a size‐selective manner. Since **P**_**Zn**_ **4EG** and **P**_**Zn**_ **5EG** both possess oligo(ethylene glycol) straps, it was expected that they could accommodate alkali metal ions as guest species. Consequently, a spectroscopic titration study was conducted to examine the alkali metal ion bindings to **P**_**Zn**_ **4EG** and **P**_**Zn**_ **5EG**. The addition of K^+^, Na^+^, and Li^+^, as the form of their PF_6_^−^ salts, to solutions of **P**_**Zn**_ **4EG** and **P**_**Zn**_ **5EG** in THF resulted in no spectral changes, indicating that alkali metal ions cannot bind to **P**_**Zn**_ **4EG** and **P**_**Zn**_ **5EG** under these solvent conditions (Figure S3). In contrast, when the solvent was changed to 10% MeCN/CHCl_3_, **P**_**Zn**_ **4EG** and **P**_**Zn**_ **5EG** showed significant spectral changes upon the addition of Li^+^ and K^+^, respectively. Furthermore, in this solvent system, the **P**_**Zn**_ **4EG** and **P**_**Zn**_ **5EG** solutions showed color change from red to green because of the alkali metal ion additions (Figure S4). Such color changes can be exploited in useful applications for the determination of metal ions using the naked eye. Interestingly, both **P**_**Zn**_ **4EG** and **P**_**Zn**_ **5EG** exhibit high selectivity toward alkali metal ions. **P**_**Zn**_ **4EG** in 10% MeCN/CHCl_3_ showed a gradual redshift in its absorption spectra only upon the addition of Li^+^ (Figure 2), while the addition of Na^+^ and K^+^ did not cause any spectral changes (Figure S5). In contrast, **P**_**Zn**_ **5EG** exhibited spectral changes only upon the addition of K^+^ (Figure 2); no appreciable change of absorption spectrum occurred because of Li^+^ or Na^+^ additions (Figure S5). The association constants observed between **P**_**Zn**_ **4EG** and **P**_**Zn**_ **5EG** and Li^+^ and K^+^ were also estimated using nonlinear curve fitting of the spectral changes utilizing HypSpec software. The association constants estimated for **P**_**Zn**_ **4EG** toward Li^+^ and **P**_**Zn**_ **5EG** toward K^+^ were 3.8 × 10^3^ and 1.0 × 10^4^ M^−1^, respectively.

### 2.4 Crystallographic and computational calculations

**FIGURE 3:**
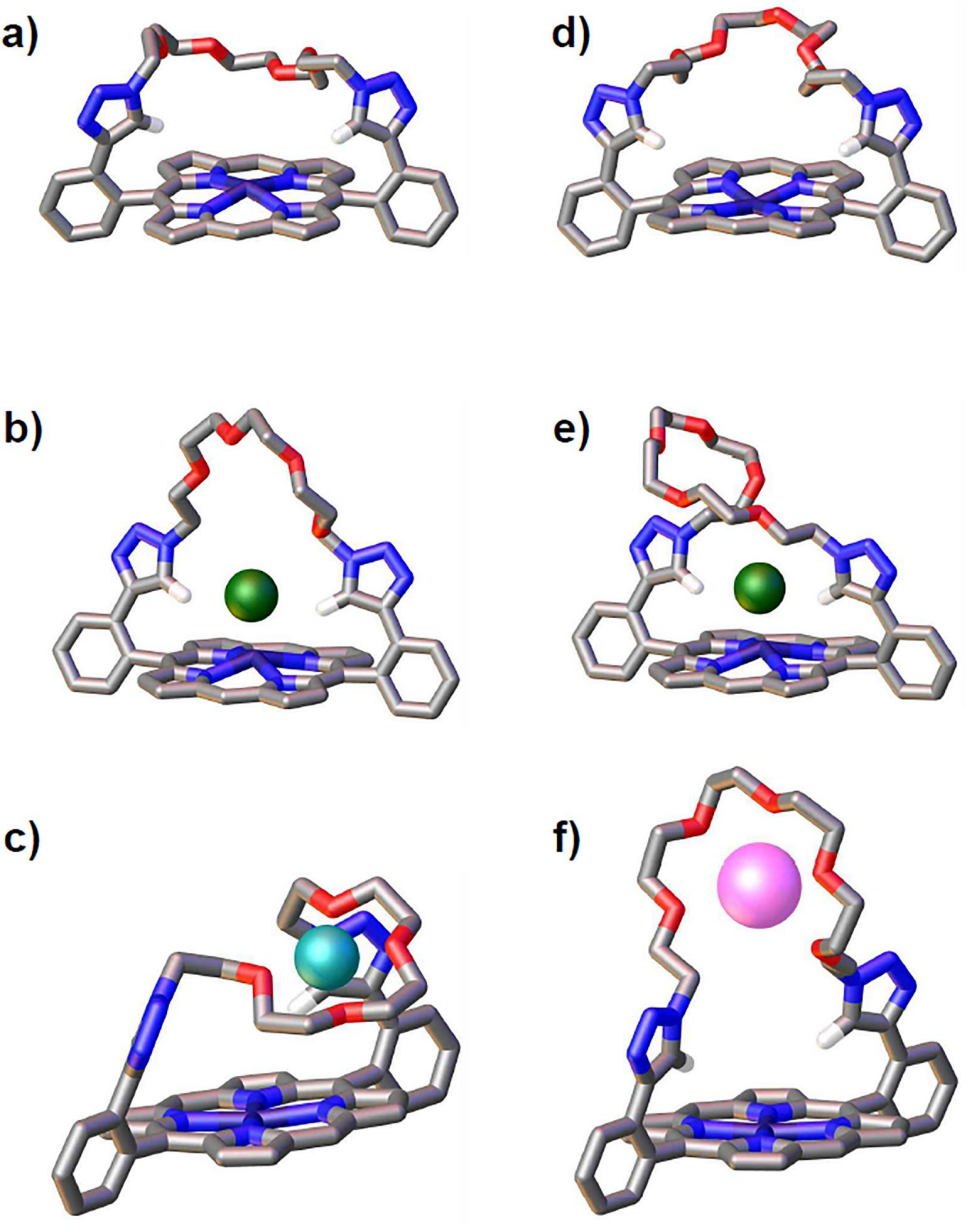
The single‐crystal structure of (a) **P**_**Zn**_ **4EG** and (d) **P**_**Zn**_ **5EG**. The energy‐minimized molecular structure of **P**_**Zn**_ **4EG** with (b) Cl^−^ and (c) Li^+^, and **P**_**Zn**_ **5EG** with (e) Cl^−^ and (f) K^+^. Solvent, H_2_O, and all hydrogens except triazole C–H were omitted for clarity.

Single‐crystal X‐ray structures were successfully obtained for both **P**_**Zn**_ **4EG** and **P**_**Zn**_ **5EG**. Energy‐minimized structural optimization was carried out for the host–guest complexes based on the single‐crystal structures using density functional theory calculations (Figure 3). The calculations were performed using Beck's three‐parameter hybrid exchange functional and the Lee–Yang–Parr correlation functional (B3LYP), employing the 6‐31G(d) basis set.^9^ The results indicate that the triazole C–H protons are directed to the porphyrin center and create a cavity for halides to form hydrogen bonds. The oligo(ethylene glycol) straps were expected to show similar binding performance as crown ethers. The results confirmed this hypothesis, showing that ethylene glycol straps capture alkali metal ions and behave like crown ethers.

## 3 CONCLUSION

A porphyrin‐based host compound having an oligo(ethylene glycol) strap connected via a picket fence‐type triazole moiety was newly designed. These new compounds can accommodate both alkali metal and halide ions as guest species. Interestingly, the binding of alkali metal and halides ions to the porphyrin‐based hosts occurs in a solvent‐dependent manner. In THF, the host compounds can only accommodate halide ions, whereas they only accommodate alkali metal ions in 10% MeCN/CHCl_3_. Moreover, the host compounds exhibit selective binding toward alkali metal ions. **P**_**Zn**_ **4EG** with a small glycol strap can only accommodate Li^+^, but **P**_**Zn**_ **5EG**, which has a large strap, can only accommodate K^+^ as a guest species. Since the binding of alkali metal ions to the host compounds causes a significant color change, these triazole‐bearing oligo(ethylene glycol)‐strapped porphyrin derivatives are expected to be useful materials for the alkali metal ion determination.

## CONFLICT OF INTEREST STATEMENT

The authors declare no conflicts of interest.

## Supporting information

Supporting Information S1

## Data Availability

The data that support the findings of this study are available from the corresponding author upon reasonable request.
