# Supplementary material for: Triazole‐bearing oligo(ethylene glycol)‐strapped zinc porphyrins as dual mode ion‐binding receptors
Source: Smart Mol. 2023 Mar 20;1(2):e20220004. doi: 10.1002/smo.20220004 (PMC12118295; doi:10.1002/smo.20220004)
Supplement: Supplementary file 1 — Supporting Information S1 [file SMO2-1-e20220004-s001.docx]

Supporting Information

Triazole-bearing oligo(ethylene glycol)-strapped zinc porphyrins as dual mode ion-binding receptors

Jeong Heon Lee, Kyeong-Im Hong, Woo Hyeok Choi, Younghun Kim and Woo-Dong Jang*

Materials and measurements

Reagent grade was used for all commercially available reagents without further purification. Before each use solvents for titration study, all solvents were freshly distilled. Tetrabutylammonium salts of anions used for NMR experiment. ^1^H, ^13^C NMR experiments were conducted using a Bruker DPX 400 spectrometer at 25 °C. UV/Vis absorption was measured using a JASCO model V-660 spectrometer. MALDI-TOF-MS was performed on a Bruker Daltonics LRF20 with dithranol as the matrix.

Synthesis

**2**: 2-bromobenzaldehyde (10.0 g, 54.0 mmol), PdCl_2_(PPh_3_)_2_ (0.758 g, 1.08 mmol), and CuI (0.206 g, 1.08 mmol) were added in 2-necked, 250 mL round bottomed flask. The mixture was vacuumed, and added 100 mL of Et_3_N and trimethylsilylacetylene (10.6 g, 108 mmol) to the flask under N_2_ atmosphere. The solution was stirred at 80 °C for 12 hr. To remove Et_3_N, the reaction mixture was poured into CH_2_Cl_2_ (100 mL) and washed with distilled water 3 times. Then, the combined organic layer was concentrated under reduced pressure, and purified by silica column chromatography using EtOAc:Hexane (1:10) as a eluent to yield **2** as a yellowish white solid (9.01 g, 44.5 mmol, 82%). ^1^H NMR (400 MHz, CDCl_3_, *δ*): 10.56 (s, 1 H), 7.93-7.88 (m, 1 H), 7.58-7.51 (m, 2 H), 7.45-7.41 (m, 1 H), 0.28 (s, 9 H).

**3**: Dipyrrolomethane (2.0 g, 13.7 mmol), trifluoroacetic acid (1.0 mL, 13.1 mmol), and **2** (2.77 g, 0.137 mmol) were dissolved in CH_2_Cl_2_ (800 mL) and stirred for 3 hr. After the addition of Et_3_N (2.0 mL, 14.2 mmol) and *p*-chloranil (6.74 g, 27.4 mmol), the reaction mixture was further stirred for 4 hr. Then, the solvent was removed under reduced pressure, and purified roughly by passing through short silica column as an eluent of CH_2_Cl_2_. The crude products were dissolved in 10% MeOH/CH_2_Cl_2_, and ZnOAc (6.0 g, 27.4 mmol) was added and stirred for 3 hr. Then the solution was evaporated and purified by silica column chromatography using CH_2_Cl_2_:hexane (3:7) as an eluent to yield **3** as a purple solid (0.69 g, 0.96 mmol, 14%). ^1^H NMR (400 MHz, CDCl_3_, *δ*): 10.32 (s, 2 H), 9.42 (d, 4 H, *J* = 5.0 Hz), 9.08 (d, 4 H, *J* = 5.0 Hz), 8.24-8.21 (m, 2 H), 7.96-7.93 (m, 2 H), 7.79-7.75 (m, 4 H), -1.25 (s, 18 H). MALDI-TOF-MS: *m/z*: calcd. for C_42_H_36_N_4_Si_2_Zn:718.18 [M] ^+^; found:716.52.

**4**: To a anhydrous THF (50 mL), (0.69 g, 0.96 mmol), tetrabutylammonium fluoride (5.0 mL, 1 M in THF) was added and stirred for 40 min. The solution was concentrated and washed with water. The combined organic ralyer was evaporated and purified by silica column chromatography using CH_2_Cl_2_ as an eluent to yield **4** as a purple solid (0.52 g, 0.90 mmol, 94%). ^1^H NMR (400 MHz, CDCl_3_, *δ*): 10.31 (s, 2 H), 9.425 (d, 4 H, *J* = 8.0 Hz), 9.015 (d, 4 H, *J* = 8.0 Hz), 8.19-8.17 (d, 2 H, *J* = 4.0 Hz), 7.98 (d, 2 H, *J* = 4.0 Hz), 7.80-7.76 (m, 4 H), 2.06 (s, 2 H). MALDI-TOF-MS: *m/z*: calcd. for C_36_H_20_N_4_Zn:572.10 [M] ^+^; found:572.40.

**5b**: To CH_2_Cl­_2_ (300 mL), pentaethylene glycol (5.0 g, 21.0 mmol), *p*-toluensulfonyl chloride (12.0 g, 63.0 mmol), and potassium hydroxide (5.89 g, 0.105 mol) was added and stirred for 12 h at 0 °C. The reaction mixture was passed through celite to remove solid impurities. The filtrate was washed with water twice and purified by silica column chromatography using MeOH:CH_2_Cl_2_ (5:95) as an eluent to give **5b** as colorless oil (9.18 g, 16.8 mmol, 80%). ^1^H NMR (400 MHz, CDCl_3_, *δ*): 7.79 (d, *J* = 8.3 Hz, 4 H), 7.33 (d, *J* = 8.0 Hz, 4 H), 4.14 (t, *J* = 4.8 Hz, 4 H), 3.67 (t, *J* = 4.8 Hz, 4 H), 3.61 (s, 6 H), 3.57 (s, 6 H), 2.44 (s, 6 H).

**5c**: To a solution of **5b** (5.0 g, 9.15 mmol) in EtOH (150 mL), sodium azide (1.31 g, 20 mmol) solution in distilled water (10 mL) was slowly added and refluxed for 2 d. The reaction mixture was washed with water twice and purified by silica column chromatography using MeOH:CH_2_Cl_2_ (1:9) as an eluent to give **5c** as colorless oil (2.40 g, 8.32 mmol, 91%). ^1^H NMR (400 MHz, CDCl_3_, *δ*): 3.70-3.64 (m, 16 H), 3.39 (t, *J* = 5.1 Hz, 4 H).

**6b**: To CH_2_Cl_2_ (300 mL), hexaethylene glycol (5.0 g, 17.7 mmol), *p*-toluensulfonyl chloride (12.0 g, 53.1 mmol), and potassium hydroxide (4.97 g, 88.5 mmol) was added and stirred for 12 h at 0 °C. The reaction mixture was passed through celite to remove solid impurities. The filtrate was washed with water twice and purified by silica column chromatography using MeOH:CH_2_Cl_2_ (5:95) as an eluent to give **6b** as colorless oil (8.89 g, 15.0 mmol, 85%). ^1^H NMR (400 MHz, CDCl_3_, *δ*): 7.778 (d, *J* = 8.3 Hz, 4 H), 7.37 (d, *J* = 8.1 Hz, 4 H), 4.12 (t, *J* = 4.6 Hz, 4 H), 3.64 (t, *J* = 4.7 Hz, 4 H), 3.56 (s, 8 H), 3.53 (s, 8 H), 2.48 (s, 6 H).

**6c**: To a solution of **6b** (5.0 g, 8.46 mmol) in EtOH (150 mL), sodium azide (1.21 g, 18.6 mmol) solution in distilled water (10 mL) was slowly added and refluxed for 2 d. The reaction mixture was washed with water twice and purified by silica column chromatography using MeOH:CH_2_Cl_2_ (1:9) as an eluent to give **6c** as colorless oil (2.53 g, 7.61 mmol, 90%). ^1^H NMR (400 MHz, CDCl_3_, *δ*): 3.63-3.57 (m, 20 H), 3.35-3.29 (t, *J* = 5.1 Hz, 4 H).

**P_Zn_4EG**: To a solution of **5c** (50.0 mg, 0.17 mmol) in anhydrous DMF (10 mL), **4** (100 mg, 0.17 mmol) and [Cu(CH_3_CN)_4_]PF_6_ (12.7 mg, 34.1 μmol) were added and stirred for 3 d at 70 °C under N_2_ atmosphere. Then, the mixture was concentrated under reduced pressure, and washed with distilled water and extracted with CH_2_Cl_2_. The combined organic layer was evaporated and purified by silica column chromatography using MeOH:CH_2_Cl_2_ (5:95) as an eluent to give **P_Zn_4EG** as a purple solid (34.1 mg, 0.04 mmol, 23%). ^1^H NMR (400 MHz, CDCl_3_, δ): 10.13 (s, 2 H), 9.245 (d, 4 H, *J* = 8.0 Hz), 8.945 (d, 4 H, *J* = 8.0 Hz), 8.69 (d, 2 H, *J* = 8 Hz), 7.91-7.87 (m, 4 H), 7.65 (t, 2 H, *J* = 8 Hz), 5.32 (s, 2 H), 3.32 (s, 4 H), 2.84 - 2.82 (m, 4 Hz), 1.78 (s, 4 H) 1.49 (s, 4 H). 0.8 (s, 4 H). ^13^C NMR (100 MHz, CDCl_3_, δ): 150.1, 149.6, 147.6 (147.64), 147.6 (147.59), 140.0, 136.7, 132.9, 132.4, 132.1, 128.7, 127.7, 127.6, 126.4, 122.9, 118.7, 118.6, 106.1, 69.8, 69.0, 68.8, 68.7, 68.4, 68.3, 49.9 MALDI-TOF-MS: *m/z*: calcd. for C_46_H_40_N_10_O_4_Zn:860.25 [M]^+^; found: 861.40.

**P_Zn_5EG**: To a solution of **6c** (58.0 mg, 0.17 mmol) in anhydrous DMF (10 mL), **4** (100 mg, 0.17 mmol) and [Cu(CH_3_CN)_4_]PF_6_ (12.7 mg, 34.1 μmol) were added and stirred for 3 d at 70 °C under N_2_ atmosphere. Then, the mixture was concentrated under reduced pressure, and washed with distilled water and extracted with CH_2_Cl_2_. The combined organic layer was evaporated and purified by silica column chromatography using MeOH:CH_2_Cl_2_ (5:95) as an eluent to give **P_Zn_5EG** as a purple solid (33.7 mg, 0.037 mmol, 22%). ^1^H NMR (400 MHz, CDCl_3_, δ): 10.17 (s, 2 H), 9.29 (d, *J* =4.4 Hz, 4 H), 8.98 (d, *J* = 4.4 Hz, 4 H), 8.66 (d, *J* = 7.8 Hz, 2 H), 7.94-7.88 (m, 4 H), 7.68-7.62 (m, 2 H), 5.69 (s, 2 H), 3.47 (t, *J* = 4.5 Hz, 4 H), 2.94 (t, *J* = 4.4 Hz, 4 H), 2.15 (s, 4 H), 1.59 (s, 4 H), 1.08 (s, 8 H). ^13^C NMR (100 MHz, CDCl_3_, δ): 150.2, 149.7, 147.6, 140.2, 136.2, 133.0, 132.4, 132.1, 128.7, 127.8, 126.4, 123.1, 118.7, 106.1, 69.7, 69.0, 68.8, 68.3, 49.7, 1.2, 0.2. MALDI-TOF-MS: *m/z*: calcd for C_48_H_44_N_10_O_5_Zn:906.33 [M]^+^; found: 906.80.


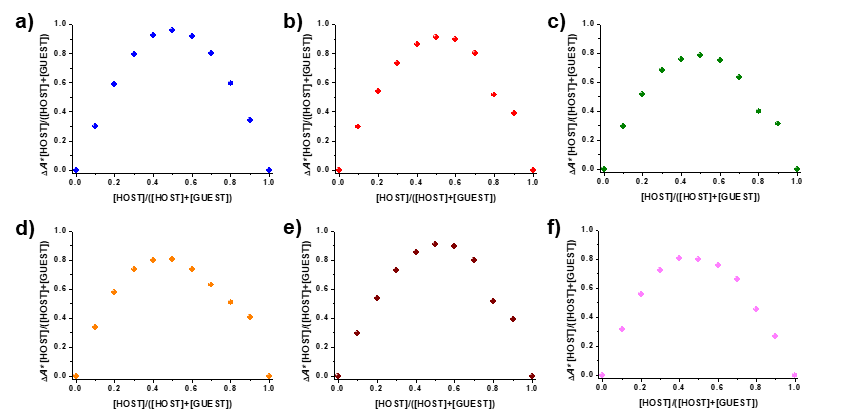
**Figure S1.** Job’s plots of **P_Zn_4EG** (a-c) and **P_Zn_5EG** (e-f) upon the addition of a, e) Cl^–^, b, d) Br^–^, and c, f) I^–^ ([Host] = 2.5 µM, THF).


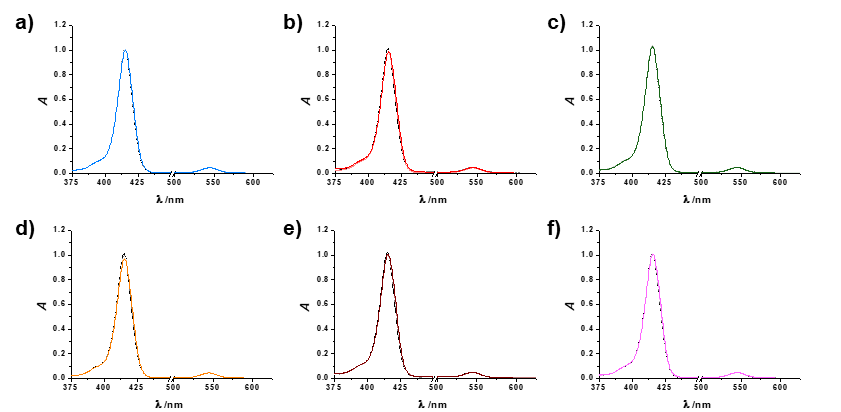


**Figure S2.** Spectroscopic changes observed **P_Zn_4EG** (a-c) and **P_Zn_5EG** (e-f) upon the addition of a, d) Cl^–^, b, e) Br^–^, and c, f) I^–^. ([Host] = 2.5 µM, [Guest] = 0 – 50 µM, 10% MeCN/CHCl_3_).


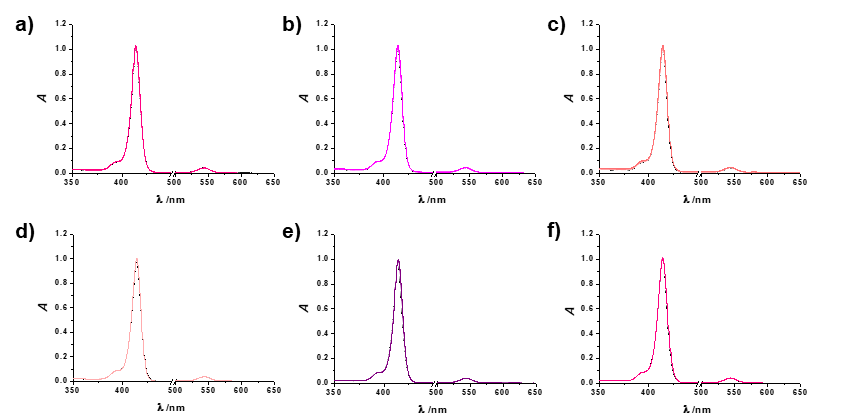
**Figure S3.** Spectroscopic changes observed for **P_Zn_4EG** (a-c) and **P_Zn_5EG** (e-f) upon the addition of a, e) Li^+^, b, d) Na^+^, and c, f) K^+^. ([Host] = 2.5 µM, [Guest] = 0 – 250 µM, 10% MeCN/CHCl_3_).


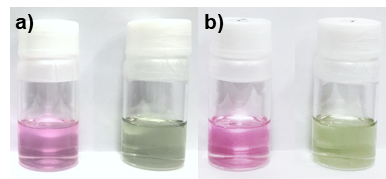


**Figure S4.** Observation of colour changes for **P_Zn_4EG** (a) and **P_Zn_5EG** (b) upon the addition of Li^+^ and K^+^ respectively. ([Host] = 5 µM, 10% MeCN/CHCl_3_).


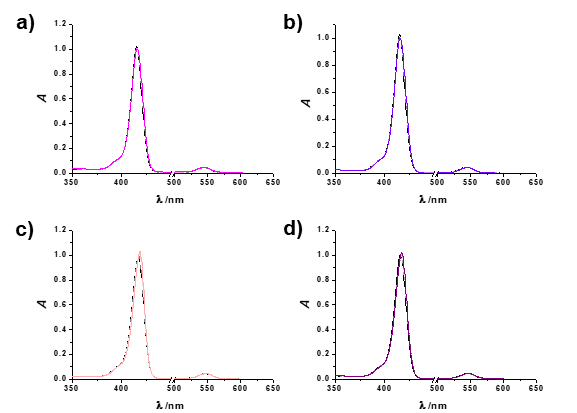


**Figure S5.** Spectroscopic changes observed for **P_Zn_4EG** (a, b) and **P_Zn_5EG** (c, d) upon the addition of a) Na^+^, b) K^+^, c) Li^+^ and d) Na^+^ ([Host] = 2.5 µM, [Guest] = 0 – 250 µM, 10% MeCN/CHCl_3_).

**Figure S6.** ^1^H NMR spectrum of **2** (CDCl_3_, 400 MHz).

**Figure** **S7** ^1^H NMR spectrum of **3** (CDCl_3_, 400 MHz).

 **Figure** **S8**. ^1^H NMR spectrum of **4** (CDCl_3_, 400 MHz).

**Figure** **S9**.^1^H NMR spectrum of **5b** (CDCl_3_, 400 MHz).

 **Figure** **S10**.^1^H NMR spectrum of **5c** (CDCl_3_, 400 MHz).

**Figure** **S11**.^1^H NMR spectrum of **6b** (CD_2_Cl_2_, 400 MHz).

 **Figure** **S12**.^1^H NMR spectrum of **6c** (CDCl_3_, 400 MHz).

**Figure** **S13**.^1^H NMR spectrum of **P_Zn_4EG** (CDCl_3_, 400 MHz).

 **Figure** **S14**.^13^C NMR spectrum of **P_Zn_4EG** (CDCl_3_, 100 MHz).

**Figure** **S15**.^1^H NMR spectrum of **P_Zn_5EG** (CDCl_3_, 400 MHz).

 **Figure** **S16**.^13^C NMR spectrum of **P_Zn_5EG** (CDCl_3_, 100 MHz).

**X-ray crystallographic data of P_Zn_4EG**

| **Table 1 Crystal data and structure refinement for POR_4_PEG.** | |
| --- | --- |
| Identification code | POR_4_PEG |
| Empirical formula | C_54_H_58_N_10_O_8_Zn |
| Formula weight | 1040.47 |
| Temperature/K | 100 |
| Crystal system | monoclinic |
| Space group | Pn |
| a/Å | 12.430(3) |
| b/Å | 12.354(3) |
| c/Å | 16.516(3) |
| α/° | 90 |
| β/° | 103.39(3) |
| γ/° | 90 |
| Volume/Å^3^ | 2467.2(9) |
| Z | 2 |
| ρ_calc_g/cm^3^ | 1.401 |
| μ/mm^‑1^ | 0.774 |
| F(000) | 1092.0 |
| Crystal size/mm^3^ | 0.126 × 0.07 × 0.06 |
| Radiation | synchrotron (λ = 0.800) |
| 2Θ range for data collection/° | 3.71 to 55.996 |
| Index ranges | -14 ≤ h ≤ 14, -13 ≤ k ≤ 14, -19 ≤ l ≤ 19 |
| Reflections collected | 7741 |
| Independent reflections | 7740 [R_int_ = 0.0426, R_sigma_ = 0.0384] |
| Data/restraints/parameters | 7740/14/657 |
| Goodness-of-fit on F^2^ | 1.048 |
| Final R indexes [I>=2σ (I)] | R_1_ = 0.0553, wR_2_ = 0.1433 |
| Final R indexes [all data] | R_1_ = 0.0568, wR_2_ = 0.1444 |
| Largest diff. peak/hole / e Å^-3^ | 1.52/-0.85 |
| Flack parameter | 0.490(5) |

**Data Collection**

The diffraction data from red crystals of PZn4EG (0.126 × 0.07 × 0.06 mm3) mounted on a MiTeGen MicroMount© were collected at 100 on a ADSC Quantum 210 CCD diffractometer with synchrotron radiation (0.8000 Å) at Supramolecular Crystallography 2D, Pohang Accelerator Laboratory (PAL), Pohang, Korea. The ADSC Q210 ADX program^1^ was used for data collection (detector distance is 63 mm, omega scan; Δω = 3º, exposure time is 1 sec/frame for PZn4EG and HKL3000sm (Ver. 703r)^2^ was used for cell refinement, reduction and absorption correction. The crystal structures of PZn4EG was solved by the direct method with SHELX-XT (Ver. 2014/5)^3^ and refined by full-matrix least-squares calculations with the SHELX-XL (Ver. 2016/4)^4^ program package.

**Structure Solution and Refinement**

The systematic absences in the diffraction data were uniquely consistent for the monoclinic space group Pn that yielded chemically reasonable and computationally stable results of refinement^4-5^. A successful solution by the direct methods provided most non-hydrogen atoms from the E-map. The remaining non-hydrogen atoms were located in an alternating series of least-squares cycles and difference Fourier maps. All non-hydrogen atoms were refined with anisotropic displacement coefficients. All hydrogen atoms were included in the structure factor calculation at idealized positions and were allowed to ride on the neighboring atoms with relative isotropic displacement coefficients. The final least-squares refinement of 657 parameters against 7740 data resulted in residuals R (based on F2 for I≥2σ) and wR (based on F2 for all data) of 0.0553 and 0.1444, respectively. The final difference Fourier map was featureless.

**Summary**

**Crystal Data for C_54_H_58_N_10_O_8_Zn (M =1040.47 g/mol):** monoclinic, space group Pn (no. 7), a = 12.430(3) Å, b = 12.354(3) Å, c = 16.516(3) Å, β = 103.39(3)°, V = 2467.2(9) Å3, Z = 2, T = 100 K, μ(synchrotron) = 0.774 mm-1, Dcalc = 1.401 g/cm3, 7741 reflections measured (3.71° ≤ 2Θ ≤ 55.996°), 7740 unique (Rint = 0.0426, Rsigma = 0.0384) which were used in all calculations. The final R1 was 0.0553 (I > 2σ(I)) and wR2 was 0.1444 (all data).

**X-ray crystallographic data of P_Zn_5EG**

| **Table 1 Crystal data and structure refinement for POR_5_PEG.** | |
| --- | --- |
| Identification code | POR_5_PEG |
| Empirical formula | C_49_H_47_Cl_3_N_10_O_6_Zn |
| Formula weight | 1043.68 |
| Temperature/K | 305.1 |
| Crystal system | triclinic |
| Space group | P-1 |
| a/Å | 9.4998(5) |
| b/Å | 11.4492(6) |
| c/Å | 23.0055(13) |
| α/° | 97.002(2) |
| β/° | 93.052(2) |
| γ/° | 91.563(2) |
| Volume/Å^3^ | 2478.6(2) |
| Z | 2 |
| ρ_calc_g/cm^3^ | 1.398 |
| μ/mm^‑1^ | 0.716 |
| F(000) | 1080.0 |
| Crystal size/mm^3^ | 0.15 × 0.12 × 0.1 |
| Radiation | MoKα (λ = 0.71073) |
| 2Θ range for data collection/° | 4.2 to 52.288 |
| Index ranges | -11 ≤ h ≤ 11, -14 ≤ k ≤ 14, -28 ≤ l ≤ 28 |
| Reflections collected | 84285 |
| Independent reflections | 9867 [R_int_ = 0.0718, R_sigma_ = 0.0383] |
| Data/restraints/parameters | 9867/115/731 |
| Goodness-of-fit on F^2^ | 1.045 |
| Final R indexes [I>=2σ (I)] | R_1_ = 0.0665, wR_2_ = 0.1713 |
| Final R indexes [all data] | R_1_ = 0.0916, wR_2_ = 0.1896 |
| Largest diff. peak/hole / e Å^-3^ | 1.53/-1.00 |

**Data Collection**

A crystal with approximate dimensions 0.1 × 0.12× 0.15 mm3 was selected under oil under ambient conditions and attached to the tip of a MiTeGen MicroMount©. The crystal was mounted and centered in the X-ray beam by using a video camera. The crystal evaluation and data collection were performed on a Bruker D8 Venture diffractometer with Mo Kα (λ = 0.71073 Å) radiation and the diffractometer to crystal distance of 4.00 cm. The initial cell constants were obtained from three series of ω scans at different starting angles. Each series consisted of 12 frames collected at intervals of 0.5º in 6 range about ω with the exposure time of 10 seconds per frame. The reflections were successfully indexed by an automated indexing routine built in the APEXII program. The final cell constants were calculated from a set of 9910 strong reflections from the actual data collection. The data were collected by using the full sphere data collection routine to survey the reciprocal space to the extent of a full sphere to a resolution of 0.81 Å. A total of 84285 were harvested by collecting 9 set of frames with 0.5º scans in ω and φ with an exposure time 10 sec per frame. These highly redundant datasets were corrected for Lorentz and polarization effects. The absorption correction was based on fitting a function to the empirical transmission surface as sampled by multiple equivalent measurements.^1^

**Structure Solution and Refinement**

The systematic absences in the diffraction data were uniquely consistent for triclinic, space group P-1 (no. 2) that yielded chemically reasonable and computationally stable results of refinement^4-5^. A successful solution by the direct methods provided most non-hydrogen atoms from the E-map. The remaining non-hydrogen atoms were located in an alternating series of least-squares cycles and difference Fourier maps. All non-hydrogen atoms were refined with anisotropic displacement coefficients. All hydrogen atoms were included in the structure factor calculation at idealized positions and were allowed to ride on the neighboring atoms with relative isotropic displacement coefficients. The final least-squares refinement of 731 parameters against 9867 data resulted in residuals R (based on F2 for I≥2σ) and wR (based on F2 for all data) of 0.0665 and 0.1896, respectively. The final difference Fourier map was featureless.

**Summary**

**Crystal Data for C_49_H_47_Cl_3_N_10_O_6_Zn (M =1043.68 g/mol)**: triclinic, space group P-1 (no. 2), a = 9.4998(5) Å, b = 11.4492(6) Å, c = 23.0055(13) Å, α = 97.002(2)°, β = 93.052(2)°, γ = 91.563(2)°, V = 2478.6(2) Å3, Z = 2, T = 305.1 K, μ(MoKα) = 0.716 mm-1, Dcalc = 1.398 g/cm3, 84285 reflections measured (4.2° ≤ 2Θ ≤ 52.288°), 9867 unique (Rint = 0.0718, Rsigma = 0.0383) which were used in all calculations. The final R1 was 0.0665 (I > 2σ(I)) and wR2 was 0.1896 (all data).

References

(1) Arvai, A. J.; Nielsen, C. ADSC Quantum-210 ADX Program, Area Detector System

Corporation: Poway, CA, USA, 1983.

(2) Otwinowski, Z.; Minor, W. Methods in Enzymology; Carter Jr., C. W. Jr.; Sweet, R. M., Eds.; Academic Press: New York, 1997, vol. 276, part A, pp. 307-326.

(3) Sheldrick, G. M. Acta Cryst. 2015, A71, 3-8.

(4) Sheldrick, G. M. Acta. Cryst. 2015, C71, 3-8.

(5) Dolomanov, O. V.; Bourhis, L. J.; Gildea, R. J.; Howard, J. A. K.; Puschmann, H. J. Appl. Cryst. 2009, 42, 339-341.
